# Supplementary material for: Disruption of bacteriophage integration site promotes rapid diversification of multicellular traits in Bacillus subtilis
Source: Microbiol Spectr. 2026 Apr 20;14(6):e02504-25. doi: 10.1128/spectrum.02504-25 (PMC13228054; doi:10.1128/spectrum.02504-25)
Supplement: Supplemental material — Fig. S1 to S8. [file spectrum.02504-25-s0001.docx]

# SUPPLEMENTARY INFORMATION


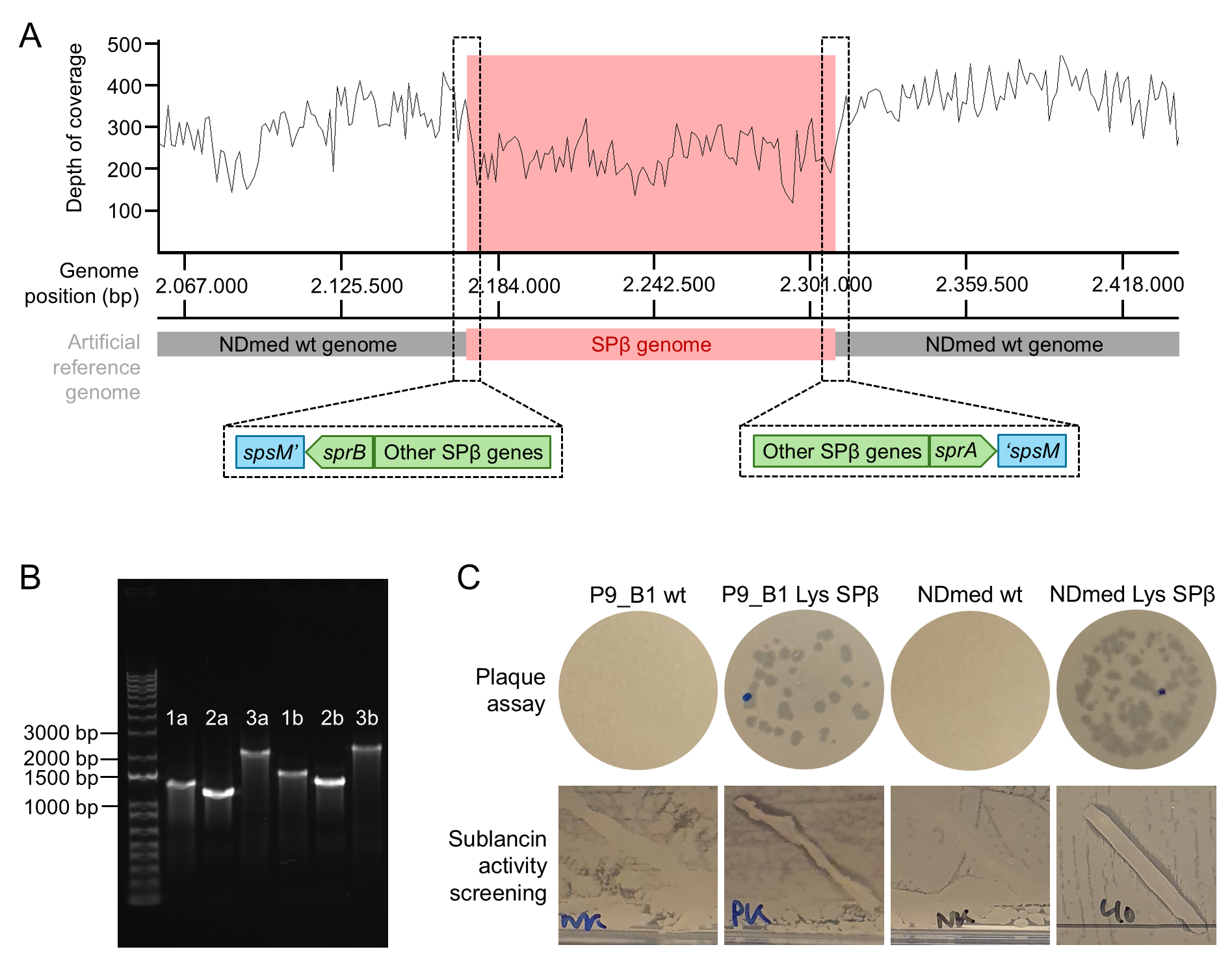


**Figure S1**. Confirmation of SPβ prophage integration into the *spsM* locus in NDmed and P9_B1 Lys SPβ strains. **A** Illumina sequencing reads from the NDmed Lys SPβ strain were aligned to an artificial reference genome in which the SPβ prophage sequence (NCBI Reference Sequence: NZ_CP045821.1) was inserted into the NDmed wild-type genome (GenBank accession: CP183238) at the expected *spsM* insertion site. The artificial genome was constructed in SnapGene. Read quality of Illumina reads was assessed using MultiQC, and reads were mapped to the artificial reference using Bowtie and SAMtools. Visualization of alignment coverage was performed in Artemis. The top panel shows the depth of coverage across the artificial genome. The lower schematic indicates genome structure, with the SPβ region highlighted in red and the flanking integration sites expanded to show the local gene organization. **B** PCR confirmation of SPβ integration in NDmed Lys SPβ and P9_B1 Lys SPβ strains. Letters “a” and “b” denote the NDmed and P9_B1 backgrounds, respectively. Lanes marked “1” show amplification of the left junction between *spsM* and SPβ, lanes “2” show the right junction, and lanes “3” indicate amplification of the sublancin gene cluster, a marker specific to the SPβ prophage. **C** Functional confirmation of SPβ prophage activity in lysogenized strains. Top row: Plaque assay showing SPβ phage release after mitomycin C induction. The appearance of plaques in the supernatant confirms the presence of inducible, active prophage. Bottom row: Sublancin-mediated antagonism assay. A 1:100 diluted lawn of the wild-type P9_B1 or NDmed strain was overlaid with a line of the lysogenized test strain. The presence of a clear inhibition zone around the line indicates sublancin production by SPβ-positive strains, confirming prophage functionality.


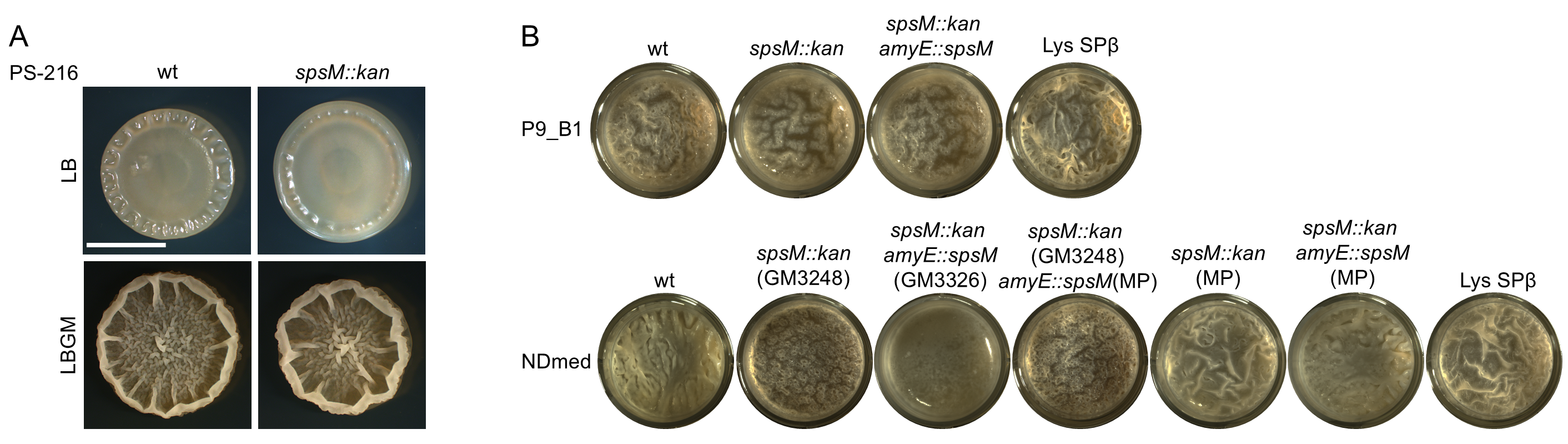


**Figure S2**. **Macrocolony biofilm of PS-216 strains and pellicle morphology of *B. subtilis* P9_B1 and NDmed strains. A** Macrocolony biofilm morphology of PS-216 strains grown on LB and LBGM media at 30°C for 48 hours. Scale bar: 5mm **B** Pellicle biofilm morphology of P9_B1 and NDmed strains grown statically in liquid LBGM medium at 30°C for 48 hours.


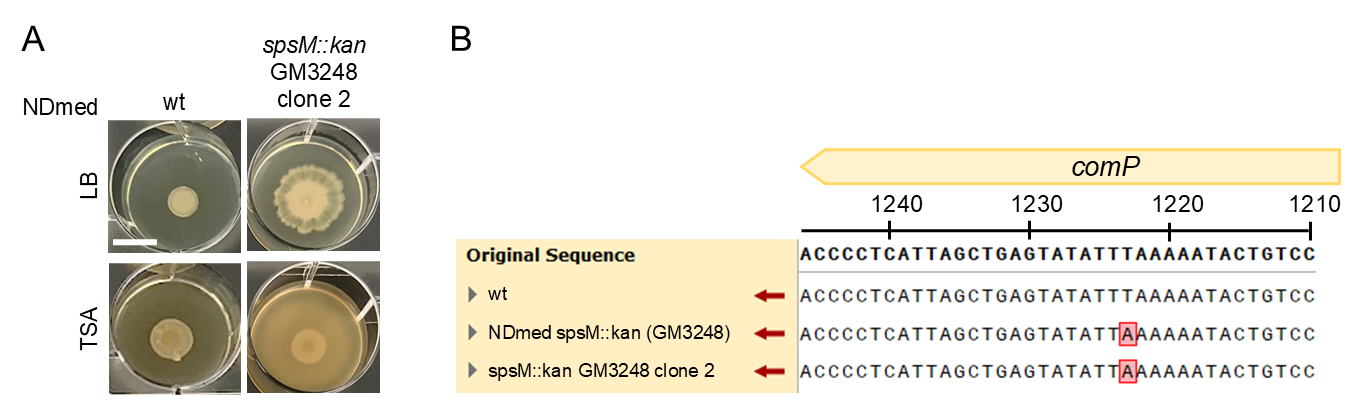


**Figure S3**. **Macrocolony morphology and *comP* gene sequences of original NDmed *spsM::kan* mutant stock. A** Macrocolony biofilm morphology of original NDmed *spsM::kan* GM3248 clone 2 stock grown on LB and TSA media at 30 °C for 5 days. Scale bar: 10 mm. **B** Sanger sequencing of the targeted region in the *comP* gene. The top panel shows a schematic representation of the wild-type *comP* reference sequence (“Original Sequence”). Below, Sanger sequencing results from each strain are aligned and compared to the reference. Deviations from the wild-type sequence are highlighted with a red box. NDmed spsM::kan (GM3248) label refers to the bacterial stock stored at Biotechincal faculty, University of Ljubljana, and spsM::kan GM3248 clone 2 label refers to the original bacterial stock made at the Micalis Institute (Sanchez-Vizuete et al., 2015).


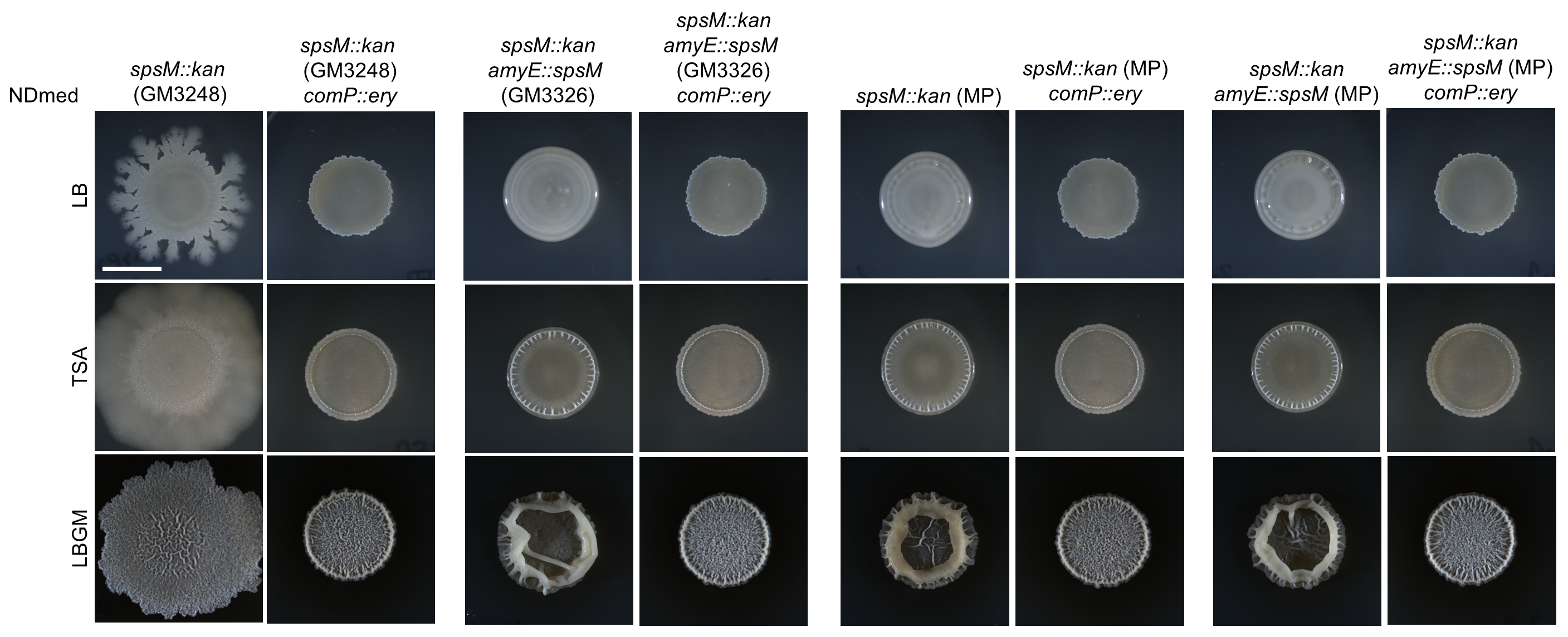


**Figure S4**. **Macrocolony morphology of NDmed strains with and without *comP::ery* deletion.** Macrocolonies were grown on LB, TSA, and LBGM media at 30°C for 48 hours. Scale bar: 5 mm


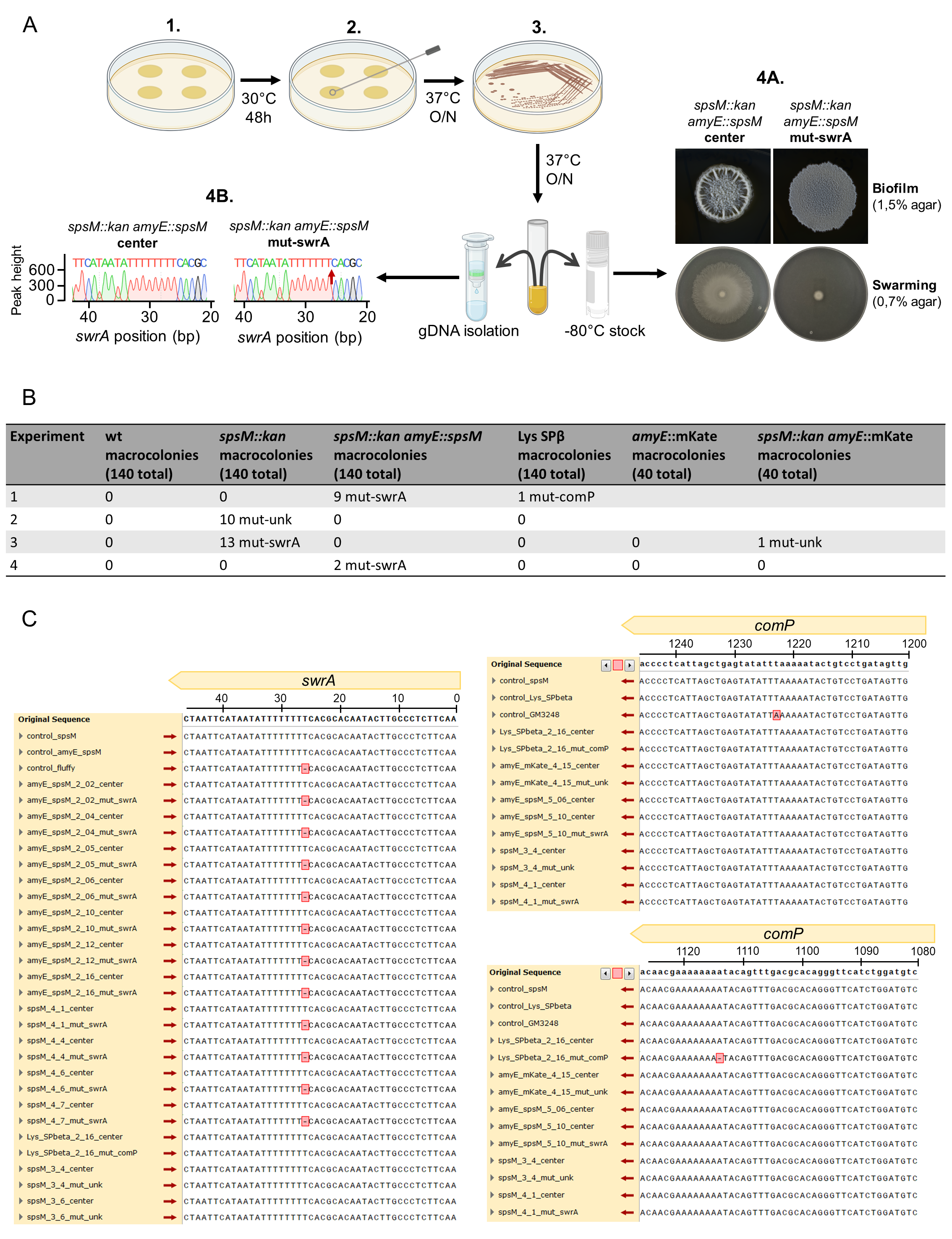


**Figure S5. Experimental workflow and results of spontaneous mutation screening in strains with active or disrupted *spsM*.** A Schematic overview of the experimental setup. All strains were inoculated on the same LB agar plate, with each plate representing one biological replicate (1). Macrocolonies were incubated for 48 hours at 30 °C. If peripheral outgrowths appeared, both the center and outgrowth of the same macrocolony were transferred to fresh LB plates (2). After further incubation, colony morphology was compared (3). Outgrowths with altered morphology were considered potential spontaneous mutants. A single colony from both the center and the outgrowth was selected to prepare –80 °C glycerol stocks and isolate genomic DNA (gDNA). Stocks were later used to assess biofilm morphology and motility phenotypes (4A), and the gDNA was used to amplify the *swrA* and *comP* genes for Sanger sequencing (4B). **B** Summary of number of outgrowths with altered morphology across four independent experiments, classified by originating strain and phenotype classification. Only a subset of mutants were sequenced (C). Nevertheless, all sequenced isolates sharing a morphological phenotype were confirmed to harbor identical mutations, validating the use of phenotype-based classification. Strains labeled mut-swrA showed colony morphology identical to those confirmed to carry the *swrA* c.26delT deletion. The single mut-comP strain carried the *comP* c.1115delT deletion. The mut-unk phenotype was characterized by a glossy colony morphology and altered swarming dynamics; however, the specific underlying mutation remains unidentified. **C** Sanger sequencing data of targeted regions in the swrA and comP genes. At the top of each aligment, schematic representations indicate the wild-type reference sequences (labeled as "Original Sequence"). Below are aligned nucleotide sequences from each analyzed strain. Strains are labeled according to their origin: colonies derived from the central region of macolonies are labeled "center," and colonies from peripheral outgrowths are labeled "mut-". Background genotypes are indicated as spsM (*spsM::kan*), amyE_spsM (*spsM::kan amyE::spsM*), and Lys_SPbeta (Lys SPβ). Strains labeled "control" represent the original parental stocks utilized for mutation screening. Additionally, sequences from the fluffy and GM3248 strains are presented as controls, representing the initial mutations identified, which motivated further mutation screening efforts. Sequence deviations relative to the wild-type are highlighted by red boxes.


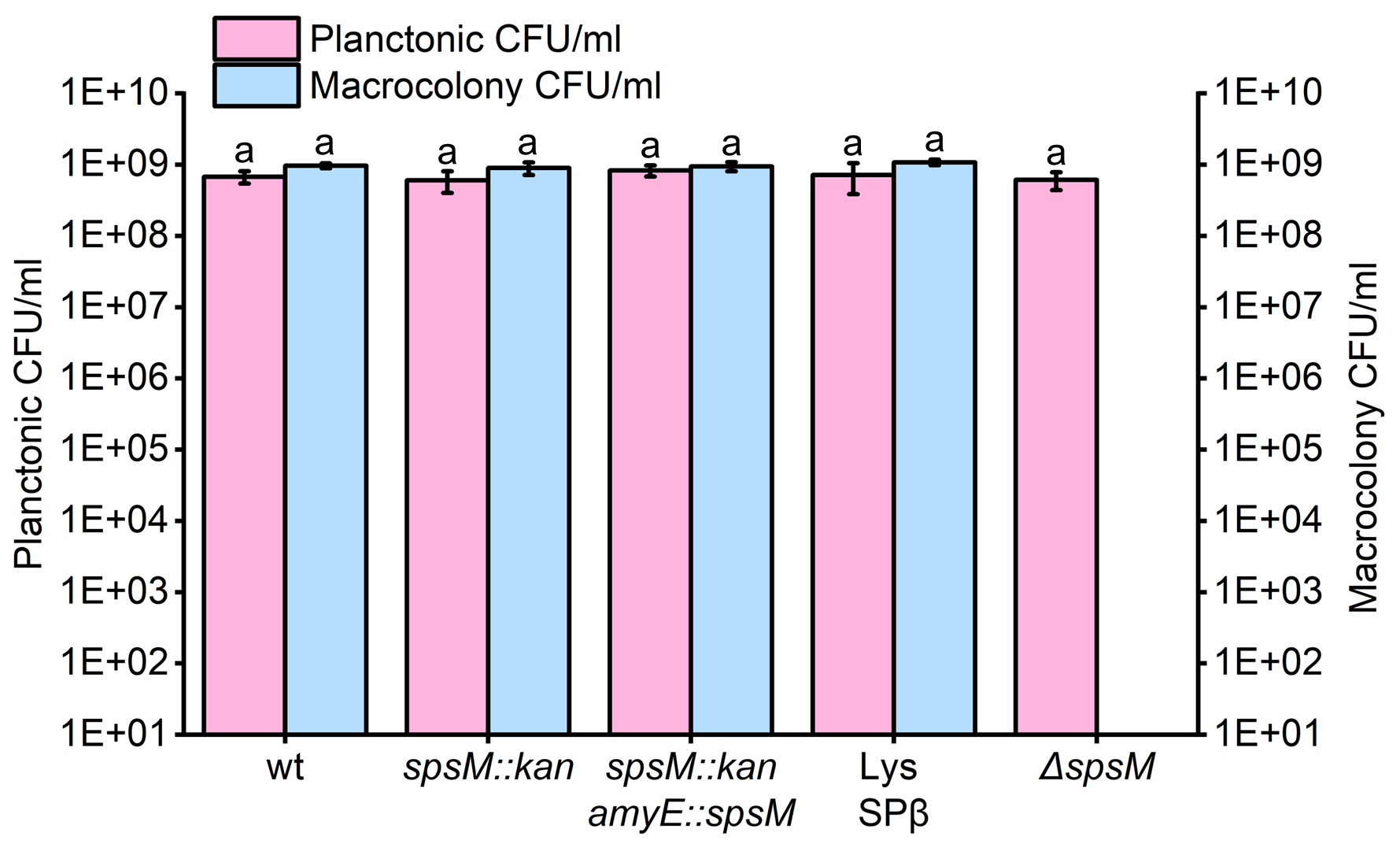


**Figure S6: Population size measurements of *B. subtilis* P9_B1 derivatives**. CFU/ml values of macrocolonies and planktonic cultures for P9_B1 wt, *spsM::kan*, *spsM::kan amyE::spsM*, SPβ lysogen and Δ*spsM* strains. Macrocolonies were grown on LB agar at 30 °C for 48 hours prior to CFU determination, whereas planktonic cultures represent the values acquired during the fluctuation assay. Columns show mean values (macrocolony: n=3; planktonic: n=6) and error bars indicate standard deviation. Statistical analysis was performed using one-way ANOVA with Tukey’s Honest Significant Difference (HSD) test. Different letters above bars denote statistically significant differences between strains (p < 0.05).


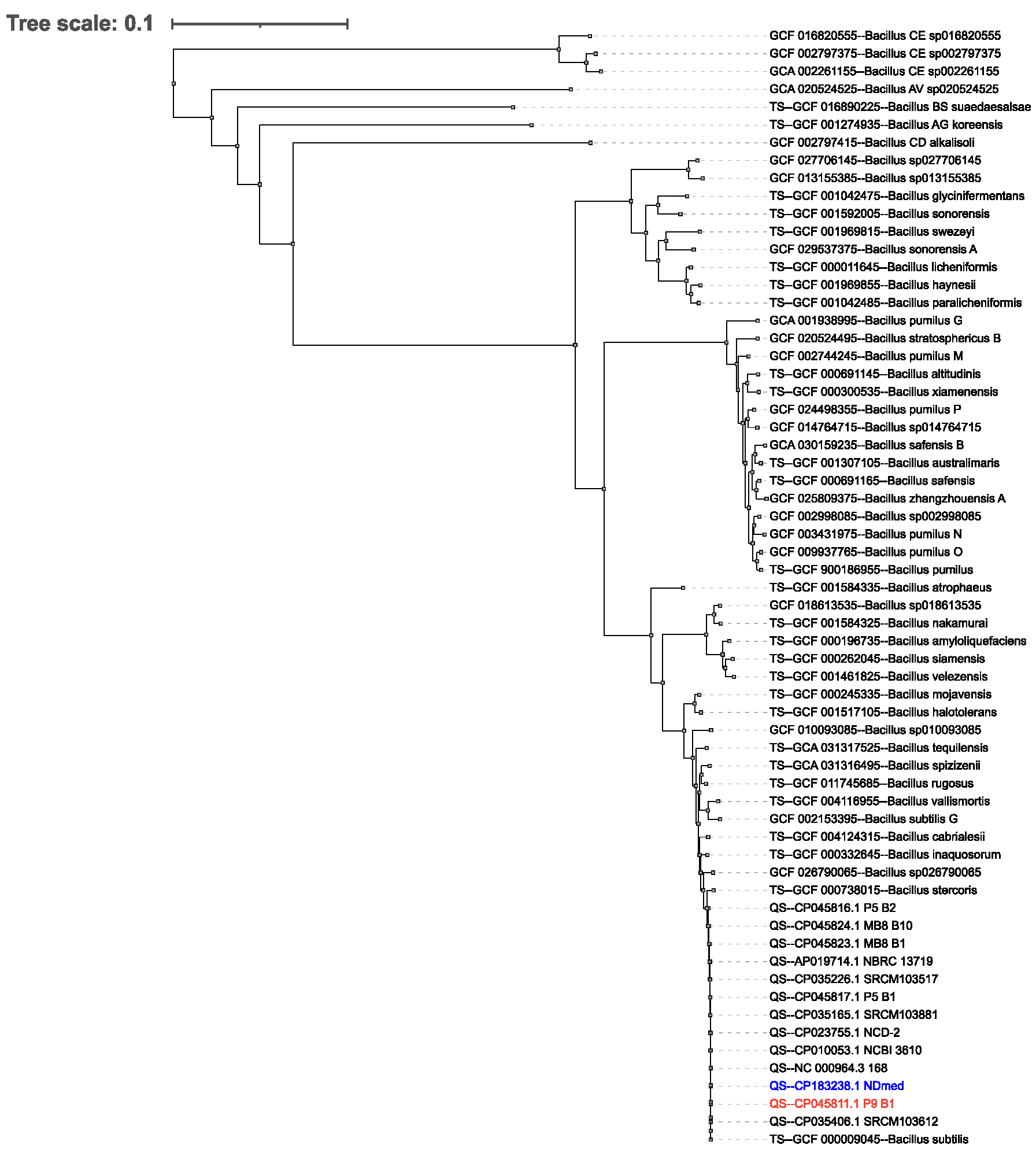


**Figure S7: Phylogenetic tree showing genentic similarity between *B. subtlis* P9_B1 (red) and *B. subtilis* NDmed (blue) compared to other closely related strains**. The tree was obtained using autoMLST2 and visualized in iTOL. Branch lengths represent substitutions per nucleotide site. The tree scale (0, 1) refers to the units of branch length displayed below the tree.


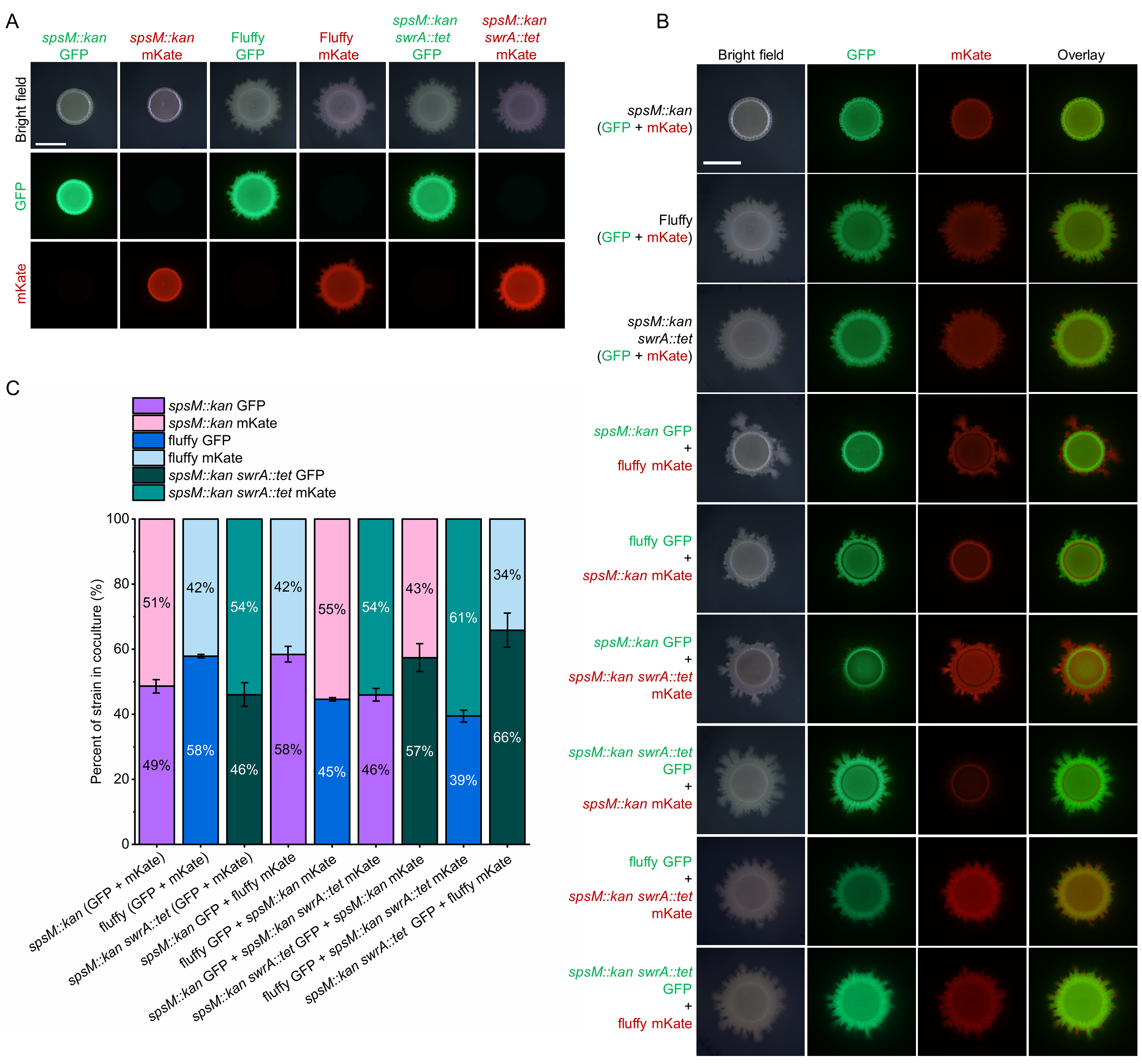


**Figure S8. Competitive coculture assays comparing *swrA*-active and *swrA*-inactive strains grown on LB medium**. All strain labels refer to P9_B1 derivatives. The fluffy strain refers to the *spsM::kan* strain carrying the spontaneous *swrA* c.26delT mutation. **A, B** Representative macrocolonies of monocultures (A) and 1:1 cocultures (B) grown on solid LB medium and incubated for 48 hours at 30 °C. Fluorescently labeled strains (GFP and mKate) were used to distinguish genotypes within each mixture. Scale bar: 5 mm. **C** Relative abundance of each strain in cocultures presented as a 100% stacked bar plot (n = 4). Fluorescence intensity was measured from macrocolony images using Fiji (ImageJ), and values were normalized using monoculture fluorescence to account for differences between GFP and mKate signal strength. Each bar shows the proportion of each strain in the coculture, reflecting its competitive fitness in biofilm-promoting conditions.
